# The Differentiation and Maintenance of SARS-CoV-2-Specific Follicular Helper T Cells

**DOI:** 10.3389/fcimb.2022.953022

**Published:** 2022-07-14

**Authors:** Yifei Wang, Qin Tian, Lilin Ye

**Affiliations:** ^1^ Guangdong Provincial Key Laboratory of Immune Regulation and Immunotherapy, School of Laboratory Medicine and Biotechnology, Southern Medical University, Guangzhou, China; ^2^ Dermatology Hospital, Southern Medical University, Guangzhou, China; ^3^ Institute of Immunology, The People’s Liberation Army (PLA), Third Military Medical University, Chongqing, China

**Keywords:** CD4^+^ T cell, follicular helper T cell, Viral infection, COVID-19, SARS-CoV-2.

## Abstract

Upon acute viral infection, virus-specific CD4^+^ T cells differentiate into either T_H_1 cells or follicular helper T (T_FH_) cells. The molecular pathways governing such bimodal cell fate commitment remain elusive. Additionally, effector virus-specific T_FH_ cells further differentiate into corresponding memory population, which confer long-term protection against re-infection of same viruses by providing immediate help to virus-specific memory B cells. Currently, the molecular mechanisms underlying the long-term maintenance of memory T_FH_ cells are largely unknown. In this review, we discuss current understanding of early differentiation of virus-specific effector T_FH_ cells and long-term maintenance of virus-specific memory T_FH_ cells in mouse models of viral infection and patients of the severe acute respiratory syndrome coronavirus 2 (SARS-CoV-2) infection.

## Introduction

**Figure 1:**
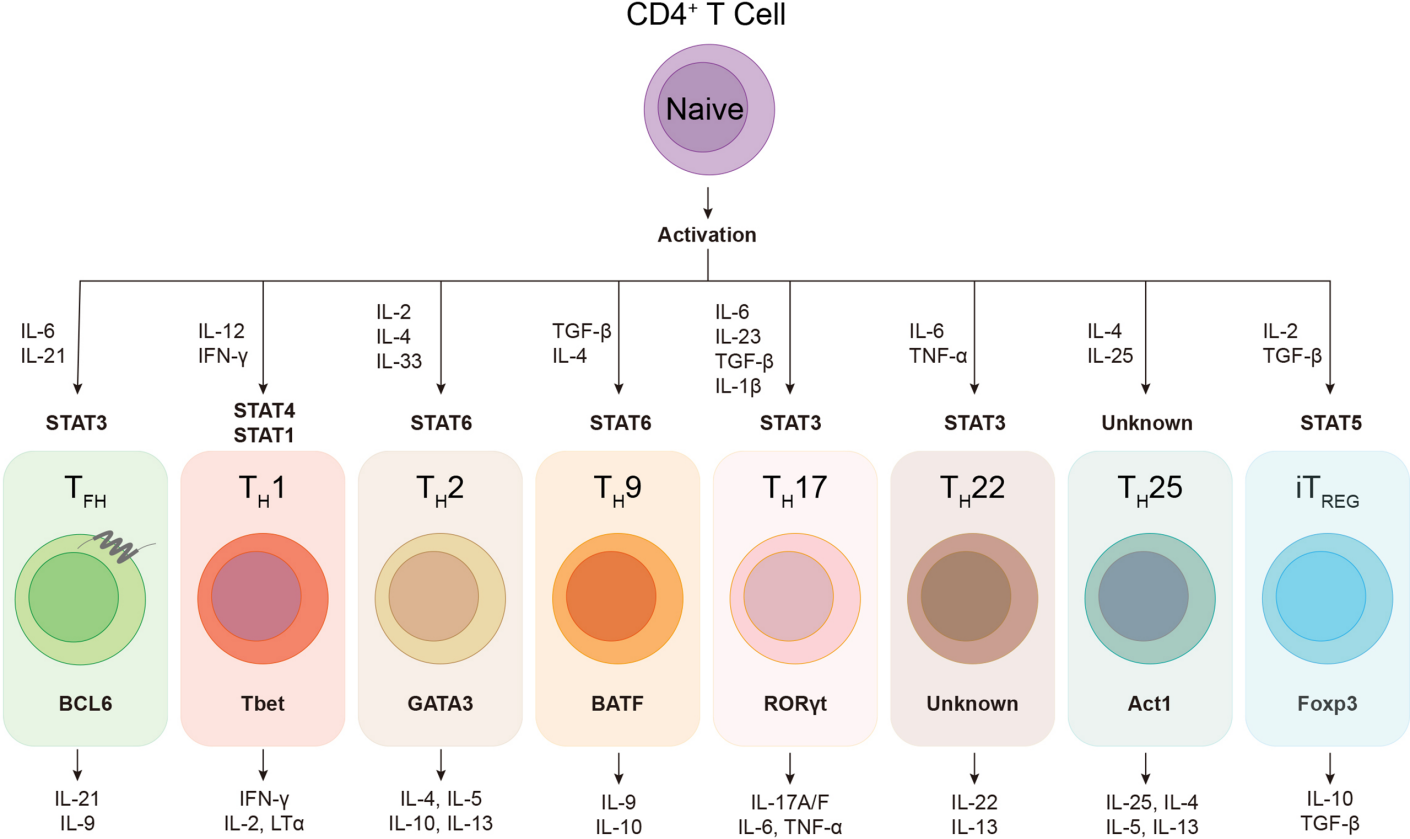
The plasticity of helper CD4+ T cells. Upon activation, naïve CD4^+^ T cell can differentiate into various subsets of T helper lineages, regulated by certain cytokines and activated signal transducers and activators of transcription (STATs). Each CD4^+^ helper lineage has the specific lineage-defining transcription factors, e.g. BCL6 in T_FH_ cell, and the characteristic profile of cytokine production, e.g. IL-21 and IL-9 in T_FH_ cell.

During viral infection, the orchestration of CD4^+^ T cells, CD8^+^ T cells and B cells constitutes the core events of host adaptive immunity, which confers specialized and long-term cellular and humoral immune protection. As “helper” cells, CD4^+^ T cells not only optimize the cytotoxic function and memory generation of CD8^+^ T cells, but also play indispensable roles in both efficient neutralizing antibody production and antibody-producing long-lived plasma cells as well as memory B cells development (Seder and Ahmed, 2003; Kurosaki et al., 2015). Regulated by specific cytokine milieu and transcriptional factors, activated CD4^+^ T cells have the potential to differentiate into various cellular subsets, including T_H_1, T_H_2, T_H_9, T_H_17, T_H_22, T_H_25, follicular helper T (T_FH_), and induced regulatory T (iT_REG_) cells **(** **Figure 1** **)**, to deal with different types of infection or non-infection situations (O’Shea John and Paul William, 2010; Caza and Landas, 2015; Das et al., 2017; Umar et al., 2020). During viral infection, virus-specific CD4^+^ T cells mainly differentiate into T helper type 1 (T_H_1) cells and follicular helper T (T_FH_) cells (Crotty, 2014; Xu et al., 2015; Huang et al., 2019). T_FH_ cell subset was first identified in human tonsils and peripheral blood, characterized by the expression of C-X-C chemokine receptor type 5 (CXCR5) and inducible costimulator (ICOS), in which the former facilitates T_FH_ cells to interact with cognate B cells and further drives B cells homing to follicles and sustains T-B interaction (Breitfeld et al., 2000; Schaerli et al., 2000).

Canonical T_FH_ cells usually locate in the intra-follicle germinal center (GC) of secondary lymphoid organs (SLO), such as lymph nodes, spleen, and tonsils, in which T_FH_ cells frequently wander out and in different GC regions, keeping close interaction with the cognate B cells (Shulman et al., 2013). GC is a highly dynamic structure where the high-affinity mutants of B cells are generated *via* somatic hypermutation (SHM) and affinity-based selection implemented by T_FH_ cells (Victora and Nussenzweig, 2012). In addition, a fraction of T_FH_ cells lingering in T-B border helps cognate B cells ahead to the extrafollicular pathways of antibody generation, which provides immediate protection against invading viruses at the early time of infection (Lee et al., 2011; Di Niro et al., 2015). In addition to SLO, T_FH_ cells have also been witnessed functional in the inducible bronchus-associated lymphoid tissues (iBALT) in lung (Tan et al., 2019), and the tertiary lymphoid structures (TLS) in tumor (Garaud et al., 2022), and the periphery circulation (Morita et al., 2011).

T_FH_ cells are featured as the key players to facilitate high-affinity antibody production of B cells *via* guaranteeing efficient SHM of immunoglobulin genes and the selective processes in GC during viral infection. After primed by dendritic cells (DCs) through engagement of viral peptide-major histocompatibility complex class II molecules (p-MHCII) complex and virus-specific TCR, T_FH_-committed CD4^+^ helper T cells initiate GC responses by moving to the T-B border and interact with cognate B cells to elicit B cell proliferation. During this process, B cells circulate between the light zone (LZ), where follicular dendritic cells (FDCs) deposit antigens and T_FH_ cells recognize the p-MHCII complexes on cognate B cells, and the dark zone (DZ), where B cells extensively proliferate after receiving “help” signals from T_FH_ cells. In DZ, GC B cells undergo rapid proliferation accompanied by SHM, allowing generation of mutated BCRs with diverse affinities to antigens. When back into LZ, mutated GC B cells with higher affinity are selected by T_FH_ cells for another circulation of proliferation and mutation (Victora and Nussenzweig, 2012; Shulman et al., 2013; Crotty, 2014). In addition to directly providing costimulatory signaling to cognate B cells *via* ICOS, CD40L, and SAP (Qi et al., 2008; Crotty, 2014), T_FH_ cells produce high levels of IL-21, which is essential for B cell survival, proliferation, plasma cell differentiation, and isotype switching (Chtanova et al., 2004; Kuchen et al., 2007; Linterman et al., 2010). In addition to IL-21, T_FH_ cell-derived IL-9 also promotes the development of memory B cells in GC (Wang et al., 2017). It was long and widely believed that T_H_1 cells rather than T_FH_ cells primarily contribute to promote killing function of CD8^+^ T cells. Of late, however, Cui et al. revealed that IL-21 produced by tumor-specific T_FH_ cells directly promotes the anti-tumor capacity of CD8^+^ T cell (Cui et al., 2021). Meanwhile, Zander et al. demonstrated that T_FH_-derived IL-21 promotes the development and antiviral immunity of CD8^+^ T cells during chronic viral infection (Zander et al., 2022). Since IL-21 promotes the formation of stem-like/memory CD8^+^ T cells (Tian and Zajac, 2016), it is possible that the help from CD4^+^ T cells to CD8^+^ T cell memory may be mediated by T_FH_ cells. Moreover, CXCR5^+^ CD4^+^ T_FH_ cells locating in perifollicular areas of iBALT act to enhance the homing and fitness of CD8^+^ T cells through IL-21 and IFN-γ production during influenza A virus infection (Pruner and Pepper, 2021).

**Figure 2:**
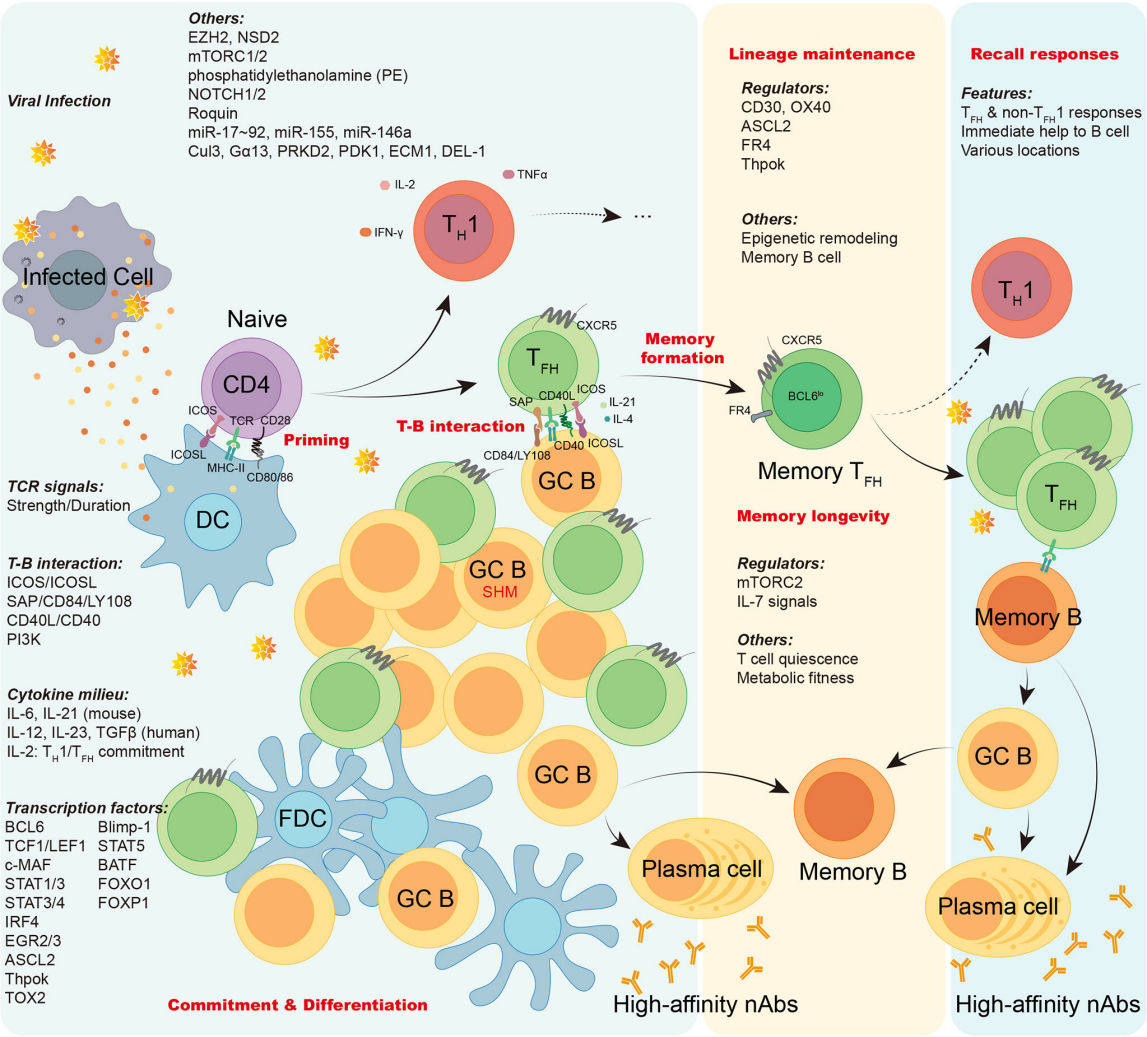
The generation, maintenance, and functions of virus-specific TFH cells. In viral infection, antigen presenting cells like dendritic cells (DCs) conduct antigen uptake, processing, and presentation to prime naïve virus-specific CD4^+^ T cells, inducing T cell activation. Upon activation, virus-specific CD4^+^ T cells are mainly committed into either T_H_1 cells or T_FH_ cells. The lineage commitment and differentiation of T_H_1/T_FH_ cell are orchestrated under the regulation of TCR signals, T-B interaction, cytokine milieu, transcription factors, and other factors as illustrated. Virus-specific effector T_FH_ cells work on initiating GC responses, facilitating the cognate B cell maturation, promoting generation of long-lived memory B cells and high-affinity neutralizing antibodies. Memory virus-specific T_FH_ cells are developed and sustained after viral clearance under the regulation of a variety of regulators, epigenetic remodeling, and cognate memory B cells. The longevity of memory T_FH_ cells are guaranteed by T cell quiescence and viability conferred by mTORC2 signaling. Upon re-challenge of encountered viruses, memory virus-specific T_FH_ cells provide immediate help to cognate memory B cells, promoting robust cellular and humoral recall responses.

**Figure 3:**
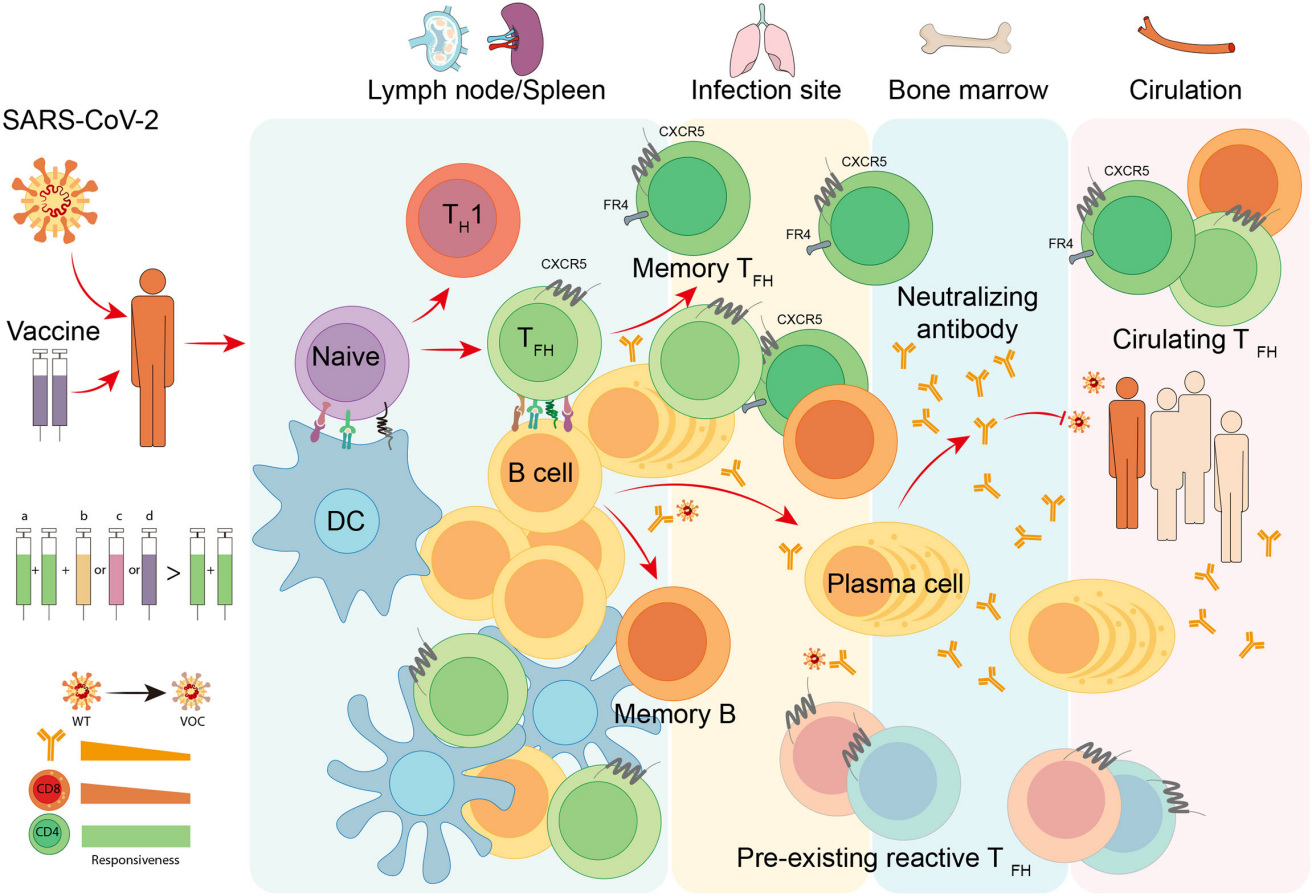
TFH cell responses to COVID-19. Infection or vaccination of SARS-CoV-2 induces differentiation of virus-specific T_FH_ cells and activation of cognate B cells in secondary lymphoid organs like lymph nodes and spleen. With the help of T_FH_ cells, antigen-specific B cells develop into plasma cells to produce neutralizing antibodies with increased affinity, and meanwhile, generate long-lived plasma cells and memory B cells that traffic to the bone marrow and provide long-term protection. Neutralizing antibodies are capable of blocking the attachment and entry of SARS-CoV-2 to prevent COVID-19. In infection site and circulation, the pre-existing cross-reactive T_FH_ cells and circulating T_FH_ cells are proved to be beneficial for the immune protection of natural infection and vaccination. To date, multiple vaccine platforms are utilized to develop SARS-CoV-2 vaccines, including (a) inactivated virus vaccines, (b) adjuvanted recombinant protein vaccines, (c) adenoviral vector-based vaccines, and (d) mRNA vaccines. The heterologous sequential immunization provides a superior effectiveness to protect vaccines from variant of concern (VOC) of SARS-CoV-2 compared to homologous vaccination. The superiority of this vaccination strategy may attribute to the competent responsiveness of memory CD4^+^ T cells to SARS-CoV-2 VOCs.

Overall, T_FH_ cells bridge the cellular and humoral immunity in host, thus playing an essential role in adaptive immune responses. Here we firstly focus on the current understanding of the generation and longevity of virus-specific T_FH_ cells during viral infection, including the fate commitment, lineage differentiation, memory formation, and long-term maintenance **(** **Figure 2** **)**. Then, we also discuss the role of SARS-CoV-2-specific T_FH_ cells during currently still ongoing pandemic coronavirus disease 2019 (COVID-19) **(** **Figure 3** **)**.

## Virus-Specific T_FH_ Cell Differentiation

### Signals from T Cell Receptor Elicit T_FH_ Commitment

T_FH_ cell differentiation is a multistep and multifactorial process. Naïve CD4^+^ T cells are primed by TCR recognition of p-MHCII presented on DCs to initiate the activation and lineage differentiation (Goenka et al., 2011). T_FH_ cell program starts at a very early stage after TCR activation. After immunization, antigen-specific CD4^+^ T cells in draining lymph nodes upregulate expression of BCL6, which is the master transcriptional factor for T_FH_ cells, within 48 hours (Baumjohann et al., 2011). Meanwhile, using lymphocytic choriomeningitis virus- (LCMV) specific TCR transgenic CD4^+^ T (SMARTA) cells, Choi et al. showed an early development of virus-specific T_FH_ cells at day 3 post-infection (Choi et al., 2011). Previous studies demonstrated that DCs are necessary and sufficient to induce CXCR5^+^ BCL6^+^ T_FH_ generation (Goenka et al., 2011), while the late B cell interactions are required for complete differentiation of T_FH_ cells (Barnett et al., 2014; Hao et al., 2018). However, in some scenarios, T_FH_ cells are generated in DC-depleted mice as long as cognate T-B interactions are available (Dahlgren et al., 2015; Arroyo and Pepper, 2019). The strength and duration of TCR signaling are considered to affect the bimodal fate commitment of T_FH_/T_H_1 cell during viral infection. By adoptive transfer of TCR transgenic T cells with different TCR affinities, Fazilleau et al. demonstrated that CD4^+^ helper T cells with higher specific binding of p-MHCII and more restricted TCR junctional diversities tend to commit to T_FH_ cell differentiation (Fazilleau et al., 2009). Further investigations suggested that the interaction between TCR and p-MHCII with long duration favors T_FH_ cell commitment (Baumjohann et al., 2013b; Tubo et al., 2013). This mechanism of T_FH_ cell commitment echoes the observation of the accumulation of T_FH_ cells during chronic viral infection, in which persistent antigen induces sustained TCR stimulation with a long dwell time (Fahey et al., 2011; Vella et al., 2017). However, there are controversial views about the facilitation of stronger TCR signals in determining T_FH_ cell commitment and differentiation (Keck et al., 2014; Snook Jeremy et al., 2018; Kotov et al., 2019). Both Keck et al. and Kotov et al. utilized the *Listeria monocytogenes* expressing peptides with different TCR affinities and corresponding TCR transgenic mice to demonstrate that TCRs with higher affinity promote T_H_1 cell formation, whereas TCRs with lower affinity poised to the T_FH_-biased differentiation of naïve CD4^+^ T cells (Keck et al., 2014; Kotov et al., 2019). In addition, Jeremy P. Snook et al. confirmed ectopically enhanced TCR signaling *via* knockdown SHP-1, which is a tyrosine phosphatase that suppresses early TCR signaling events, increases the differentiation of T_H_1 cells rather than T_FH_ cells (Snook Jeremy et al., 2018). These discrepancies warrant to be reconciled by future studies.

### ICOS, SAP, and CD40L Maintain T_FH_ Differentiation

In addition to the interaction of TCR and cognate p-MHCII on DCs, molecules associated with T-B cell conjugation, like ICOS, SAP and CD40L, are also important regulators of T_FH_ cell differentiation during viral infection (Crotty, 2011). With a high expression of CXCR5 and a low level of CCR7, T_FH_ cells are capable of migrating to the T-B border (Breitfeld et al., 2000; Schaerli et al., 2000), where the B cell-dependent T_FH_ cell differentiation occurs. ICOSL expressed on B cells is essential for the responses of T_FH_ cells (Akiba et al., 2005; Bossaller et al., 2006; Gigoux et al., 2009; Xu et al., 2013; Liu et al., 2015), in both CD28-dependent and CD28-independent pathways (Tan et al., 2006; Linterman et al., 2009). ICOSL expression on B cell is subject to the negative feedback regulation of ICOS-ICOSL interaction (Watanabe et al., 2008), while ICOS expression on T_FH_ cells seems under the negative regulation of transcriptional suppressor FOXP1 (Wang et al., 2014). ICOS signaling inactivates FOXO1, which strongly inhibits T_FH_ cell development *via* negatively regulating BCL6 expression (Stone et al., 2015). ICOS-ICOSL interaction is also required for the persistence of T_FH_ cells and GC responses by down-regulating KLF2, which serves to inhibit T_FH_ cell differentiation (Weber et al., 2015). In addition to conduct TCR signaling, PI3K signaling acts to mediate the T_FH_-promoting function of ICOS (Gigoux et al., 2009). Rolf et al. showed that the number of T_FH_ cells, GC B cells, and high-affinity antibody-secreting cells is correlated with the magnitude of PI3K signaling (Rolf et al., 2010). SAP expression on T_FH_ cell is critical for the formation of T-B interaction (Qi et al., 2008; Cannons et al., 2010a), which is indispensable for GC T_FH_ cell differentiation. Moreover, SAP actively participates in the modulation of TCR signaling in T_FH_ cells (Cannons et al., 2004; Cannons et al., 2010b). In addition, CD40L expressed on T_FH_ cells is essential for the GC B cells survival and GC maintenance as well as the function of T_FH_ cells (Elgueta et al., 2009; Crotty, 2011; Vinuesa et al., 2016).

### Cytokines Shape T_FH_ Lineage Differentiation

Cytokine milieu is pivotal to the lineage fate determination of CD4^+^ helper T cells. Unlike other subsets of CD4^+^ T cells which have the default cytokine-driving paradigm of lineage differentiation, for examples, IFN-γ and IL-12 promote T_H_1 differentiation, whereas IL-4 facilitates T_H_2 generation, T_FH_ cells manifest no default cytokine-driving differentiation pathway. Though without determining cytokines, T_FH_ cells can be shaped by multiple cytokine types (Pawlak et al., 2020).

IL-6 and IL-21 are important cytokines for T_FH_ cell differentiation in mice (Karnowski et al., 2012), whereas IL-12, IL-23, and TGF-β play prominent roles in human T_FH_ cell differentiation (Ma et al., 2009; Sweet et al., 2012; Schmitt et al., 2014). IL-21 is also an essential effector molecular of T_FH_ cells, though it can be mainly expressed by both T_H_17 and T_FH_ cells (Chtanova et al., 2004). It is generally acknowledged that IL-21 signaling is critical for the maintenance of GC in a B cell intrinsic mechanism (Linterman et al., 2010; Zotos et al., 2010). IL-6 produced by DCs induces early up-regulation of BCL6 in mouse T_FH_ cells (Eto et al., 2011), as well as promotes the maintenance of T_FH_ cells during chronic viral infection (Harker James et al., 2011). IL-21 also functions in an autocrine manner to support T_FH_ cell responses (Nurieva et al., 2008). In human, plasmacytes-derived IL-6 induces the differentiation of circulating T_FH_ cells (Chavele et al., 2015). Different with murine CD4^+^ T cells, human CD4^+^ T cells differentiate into IL-21 producing T_FH_ cells with increased expression of CXCR5, ICOS, and BCL6 under the regulation of IL-12 rather than IL-6 (Ma et al., 2009; Schmitt et al., 2009). Moreover, TGF-β, which substantially inhibits T_FH_ cell differentiation in mice, induces human T_FH_ cell development *via* activating STAT3-STAT4 signaling (Schmitt et al., 2014).

Recently, an important role of IL-2 in controlling T_FH_/non-T_FH_ cell commitment was revealed. By using IL-2 reporter mice, DiToro et al. demonstrated that naïve CD4^+^ T cells receiving highest TCR signals and producing IL-2 will differentiate into T_FH_ cells, whereas IL-2 non-producers will differentiate into non-T_FH_ cells (DiToro et al., 2018). Given abovementioned discrepant roles between certain human and mouse cytokines in regulating T_FH_ cell differentiation, whether IL-2 signaling exerts the same or similar function on mouse and human T_H_1/T_FH_ commitment needs further investigation.

### Transcription Factors Regulate T_FH_ Commitment and Differentiation

Intrinsic programs for T_FH_ cell commitment and differentiation initiate very early upon activation (Baumjohann et al., 2011; Choi et al., 2011). BCL6 is required for T_FH_ cell differentiation by inhibiting Blimp-1, which drives CD4^+^ T cells developing into non-T_FH_ lineages (Johnston Robert et al., 2009; Nurieva Roza et al., 2009; Yu et al., 2009). BCL6 expression is associated with upregulation of CXCR5 and downregulation of CCR7 and PSGL1, allowing the migration of T_FH_ cells to T-B border and GC (Hatzi et al., 2015). KLF2, another target of BCL6, impedes T_FH_ cell differentiation *via* inducing expression of *Prdm1*. *Tbx21*, and *Gata3*, and meanwhile repressing *Cxcr5* transcription *via* directly binding to its genomic region (Hatzi et al., 2015; Lee et al., 2015; Weber et al., 2015). Recent studies further elucidated that BCL6 also negatively regulates ID2 to facilitate T_FH_ cell differentiation (Shaw et al., 2016) and positively regulates TOX2 to promote chromatin accessibility of T_FH_-associated genes (Xu et al., 2019).

Since BCL6 was identified to be the master transcription factor in T_FH_ cell differentiation, plenty of transcription factors have been discovered to regulate T_FH_ cell differentiation *via* directly or indirectly affecting BCL6 expression and function (Vinuesa et al., 2016; Choi et al., 2020; Schroeder et al., 2021). TCF1 and LEF1 initiate and promote T_FH_ cell differentiation by ensuring the early expression of BCL6 and the repression of Blimp-1 (Choi et al., 2015; Wu et al., 2015; Xu et al., 2015). TCF1 is also involved in suppression of IL-2Rα (Wu et al., 2015), which together with Blimp-1 forms a negative feedback loop of TCF1/IL-2R/Blimp-1 regulating the T_FH_ responses during viral infection. In addition, signal transducers and activator (STAT) 1 and 3 both contribute to T_FH_ differentiation *via* IL-21 and IL-6 signaling (Nurieva et al., 2008). STAT3 and STAT4, in response to IL-12 and IL-23, cooperatively with TGF-β promote human but not mouse T_FH_ cell differentiation by promoting T_FH_ cell associated molecules (CXCR5, ICOS, IL-21, Bcl-6, etc.) expression and repressing Blimp-1 expression (Schmitt et al., 2014). IRF4 promotes T_FH_ cell differentiation also through signals mediated by STATs (Nurieva et al., 2008) or other transcription factors (Huber and Lohoff, 2014). However, STAT5, in collaboration with Blimp-1 and IL-2 signals, is a potent negative regulator of T_FH_ cell differentiation (Johnston et al., 2012). Also, BATF directly induces transcription of BCL6 and c-MAF in T_FH_ cell to promote the T_FH_ cell differentiation (Betz et al., 2010). Not surprisingly, absence of c-MAF decreases the amount of T_FH_ cells and IL-21 production (Bauquet et al., 2009; Andris et al., 2017). Transcription factors EGR2/3 are also required for T_FH_ cell differentiation and GC formation by regulating BCL6 (Ogbe et al., 2015). Moreover, ASCL2, which has multiple binding sites in *Cxcr5* locus, directs the migration of T_FH_ cells towards B cell follicles, and is essential for early T_FH_ cell differentiation (Liu et al., 2014). Recently, Vacchio et al. revealed that Thpok promotes BCL6 and MAF to facilitate virus-specific T_FH_ cell differentiation and GC formation in LCMV infection (Vacchio et al., 2019).

Two forkhead box proteins, FOXO1 and FOXP1, are demonstrated to negatively regulate T_FH_ cell differentiation (Wang et al., 2014; Stone et al., 2015). FOXO1 closely binds to the region of *Bcl6* locus, limiting the BCL6 expression and T_FH_ cell development (Stone et al., 2015). FOXP1 directly binds to the *Il21* promoter region to suppress IL-21 expression. In addition, FOXP1-deficient CD4^+^ T cells upregulate expression of ICOS during T cell activation (Wang et al., 2014), indicating a repressive role of FOXP1 on ICOS. In addition, a recent investigation showed that TOX2 acts to bind to and facilitate the chromatin accessibility of gene loci associated with T_FH_ cell differentiation and function, including BCL6 (Xu et al., 2019).

### Other Factors Regulating T_FH_ Cell Differentiation

Epigenetic modulation is also involved in T_FH_ cell differentiation. Besides abovementioned chromatin remodeling of T_FH_ cell associated genes *via* BCL6-TOX2 (Xu et al., 2019), the histone methyltransferase EZH2 also plays an important role in epigenetic regulation of T_FH_ cell differentiation. Using assay for transposase-accessible chromatin with high-throughput sequencing (ATAC-seq), Chen et al. demonstrated that EZH2 is essential for chromatin accessibility remodeling of T_FH_-associated genes at the early commitment of T_FH_ cells (Chen et al., 2020a). Li et al. revealed that abundant EZH2 binding peaks overlapped with TCF1 peaks, explaining the defective differentiation of T_FH_ cell with EZH2 deficiency (Li et al., 2018). Another histone methyltransferase, NSD2, which is induced by CD28 stimulation and sustained by ICOS signaling, is also required for the early expression of BCL6 and late maintenance of T_FH_ cells (Long et al., 2020).

Moreover, T cell activation and differentiation always manifest substantial re-programming of cellular metabolism (MacIver et al., 2013; Chapman et al., 2020). The serine/threonine kinase mammalian target of rapamycin (mTOR) is a potent regulator of T cell response *via* sensing and integrating inputs from nutrients, growth factors, energy, and cellular stress (Chi, 2012; Yang and Chi, 2012; Huang et al., 2020). By down-regulating mTOR, Myr-Akt, and/or CD25 signals in LCMV-specific CD4^+^ T cells, Ray et al. demonstrated that IL-2/mTORC1 axis orchestrates the reciprocal balance between T_FH_ and T_H_1 cell differentiation during viral infection (Ray et al., 2015). Further studies revealed the discrete regulatory roles of two different mTOR complexes, mTORC1 and mTORC2 (Yang et al., 2016; Zeng et al., 2016; Hao et al., 2018). Deficiency of mTORC1 substantially impairs cell proliferation and T_FH_ cell differentiation, whereas mTORC2 is needed for T_FH_ cell differentiation by promoting Akt activation and TCF1 expression without impacting cell proliferation (Yang et al., 2016). Hao et al. further demonstrated that mTORC2 signals induced by TCR and ICOS stimulation participates in cell migration, late differentiation and maturation of T_FH_ cells (Hao et al., 2018). Recently, using *in vivo* CRISPR-Cas9 screening and functional validation in mice, Fu et al. revealed a direct regulatory function of *de novo* synthesis of phosphatidylethanolamine (PE) on T_FH_ cell development *via* controlling surface expression of CXCR5 (Fu et al., 2021). Taken together, those findings highlight the metabolic control of T_FH_ cell differentiation.

In addition, these are many other factors involved in regulating T_FH_ cell commitment and differentiation. For example, RNA-binding protein Roquin exerts negative post-transcriptional regulation on T_FH_ cells *via* binding to the T_FH_-associated genes like *Icos*, *Ox40* (Vinuesa et al., 2005). Some microRNAs also play roles in post-transcriptional regulation of T_FH_ cell differentiation. miR-17~92 promotes T_FH_ cell differentiation by enhancing PI3K signaling as well as repressing non-T_FH_ genes (Baumjohann et al., 2013a). miR-155 promotes T_FH_ cell differentiation during chronic inflammation in which miR-155-knockout diminished the accumulation of T_FH_ cells (Hu et al., 2014). In addition, miR-146a acts as a post-transcriptional repressor to ICOS-ICOSL signaling and the subsequent T_FH_ cell differentiation and GC responses (Pratama et al., 2015). Also, NOTCH1/2 deficiency in CD4^+^ T cell reduces the expression of BCL6, IL-21, and CXCR5, but increases Blimp-1 expression, resulting in impairment of the development and function of T_FH_ cells. Additional factors have been verified in regulating T_FH_ cell differentiation, including but not limited to the E3 ligase cullin 3 (Cul3) (Mathew et al., 2014), heterotrimeric G protein Gα_13_ (Kuen et al., 2021), kinase PRKD2 (Misawa et al., 2020), kinase PDK1 (Sun et al., 2021), extracellular matrix protein 1 (ECM1) (He et al., 2018), and stromal cell-derived DEL-1 (Wang et al., 2021).

## Memory Virus-Specific T_FH_ Cells

### Identification of Virus-Specific Memory T_FH_ Cells

In acute viral infection or vaccination, a small proportion of the antigen-experienced CD4^+^ T cells survive after antigen clearance, subsequently become the memory CD4^+^ helper T cells. In addition to survival capacity and homeostatic proliferation without antigenic stimulation, a memory CD4^+^ helper T cell also need to preserve the lineage features during resting and recall responses (MacLeod et al., 2009; Hale and Ahmed, 2015). In the first report describing the existence of antigen-specific memory T_FH_ cells, Fazilleau et al. found a group of antigen-specific CXCR5^+^ICOS^lo^ T_FH_ cells in the memory phase of protein vaccination (Fazilleau et al., 2007). It is worth noting that those CXCR5^+^ICOS^lo^ cells were retained along with persistent peptide-MHCII (Fazilleau et al., 2007), which raises a question about the true memory property of T_FH_ cells. By adoptive transfer of TCR transgenic antigen-specific CD4^+^ T cells or T_FH_/non-T_FH_ cells into second recipients, MacLeod et al. further confirmed the existence of antigen-specific memory T_FH_ cells after protein immunization (MacLeod et al., 2011). Accumulating studies further demonstrated the validity and characteristics of virus-specific memory T_FH_ cells during viral infection and vaccination (Weber et al., 2012; Bentebibel et al., 2013; Hale et al., 2013; Locci et al., 2013).

Memory T_FH_ cells are usually marked by co-expression of CXCR5, CCR7, CD62L, and FR4, along with downregulated expression of PD1, ICOS, Ly6c, and BCL6 (Iyer et al., 2013; Hale and Ahmed, 2015). Memory T_FH_ cells exert a superior help function on naïve B cells than primary responding T_FH_ cells (MacLeod et al., 2011). In recall responses, virus-specific T_FH_ cells provide immediate help to virus-specific memory B cells (He et al., 2013; Locci et al., 2013; Phan and Tangye, 2017). In addition, local memory T_FH_ cells colocalized with B cells within the parenchymal lung tissues are critical for the production of virus-specific B cells and antibodies (Son Young et al., 2021).

### Formation of Virus-Specific Memory T_FH_ Cells

A consensus is that virus-specific memory CD4^+^ T cells are progenies of corresponding effector CD4^+^ T cells, the so-called memory precursors generated during effector phase of acute viral infection (Hale and Ahmed, 2015). But the high plasticity (Zhou et al., 2009) and non-default differentiation pathway (Choi et al., 2020) of T_FH_ cells make it hard to track a destined memory precursor T_FH_ cell at early effector phase. Some studies suggest formation of memory T_FH_ cells can be prior to GC development (He et al., 2013; Tsai and Yu, 2014). However, given the antigen retention in follicle and a rather long time of persistent GC reaction, another view is that since T_FH_ cells can shuttle between different GCs, and when they emigrate into follicles where no presented antigens exist, they acquire less activated phenotypes, resultantly, these T_FH_ cells gradually differentiate into memory cells with a resting state (Kitano et al., 2011; Choi et al., 2013).

Since T_FH_ cells are prone to stay at GC while T_H_1 cells migrate into infected location, T_FH_ cells are regarded to be more likely to differentiate into central memory T_FH_ cells (Zhu et al., 2010). Nevertheless, distribution of memory T_FH_ cells is not necessarily limited in GCs. Circulating HIV-specific effector memory T_FH_ cells are potent players for immune surveillance *in situ* (Locci et al., 2013). Moreover, iBALT-resident memory T_FH_ cells in lung are essential for the robust recall humoral responses and provide help to local CD8^+^ T_RM_ cells (Pruner and Pepper, 2021; Son Young et al., 2021).

Early study showed that CD30 and OX40 signals are needed to form CD4^+^ T cell memory (Gaspal et al., 2005). Further investigations revealed that memory T_FH_ cells down-regulate BCL6 while retain the surface expression of CXCR5 (Hale and Ahmed, 2015). ASCL2 may be important for the expression of CXCR5 in memory T_FH_ cells, because it binds to the conserved non-coding sequence regions of *Cxcr5* locus to promote *Cxcr5* transcription without inducing BCL6 expression (Liu et al., 2014). FR4, highly expressed by naïve CD4^+^ T cells, is down-regulated upon activation and re-expressed on T_FH_ cells (Iyer et al., 2013), and is maintained on memory T_FH_ cells as CXCR5 does (Hale and Ahmed, 2015). What’s more, recent study shows that long-lived T_FH_ cells persisted for over 400 days after infection are marked by high expression of FR4 (Künzli et al., 2020). Yet the mechanism driving the FR4 expression is still unknown. In addition, Ciucci et al. recently showed that Thpok is required for the signatures and emergence of memory CD4^+^ T cell *via* antagonizing the expression of Blimp-1 and Runx3 (Ciucci et al., 2019). Overall, mechanisms underlying the formation of virus-specific memory T_FH_ cells remain largely unknown. The imprinting effects from effector phase, the various features induced by different niches (GC in SLO, peripheral residence, or circulation), and driving force of the continuous expression of CXCR5, are parts of the unsolved issues about T_FH_ cell memory formation.

### Lineage Maintenance Versus Plasticity

CD4^+^ helper T cells are featured with high plasticity. Several studies showed that adoptively transferred memory T_FH_ cells eventually differentiate into both T_FH_ and non-T_FH_ cells in second recipients upon rechallenge (Pepper et al., 2011; Lüthje et al., 2012; Künzli et al., 2020). The plasticity of T_FH_ cells is highlighted by the work of Lu et al, in which the authors found that T_FH_ cells harboring chromatin accessibility of *Tbx21*, *Gata3*, and *Rorc* genes that could drive CD4^+^ Helper T cells to differentiate into T_H_1, T_H_2, and T_H_17 cells under the respective polarizing conditions (Lu et al., 2011).

Given the extrinsic factors, like antigens and effector cytokine milieu (O’Shea John and Paul William, 2010), which promote T helper cell differentiation, are absent after viral clearance, it is reasonable to consider that intrinsic programs play a dominant role in sustaining memory virus-specific T_FH_ cell lineage during memory phase. However, the master transcription factor of T_FH_ cells like BCL6, and other factors constraining T helper cell lineages are down-regulated in memory CD4^+^ T cells (Hale and Ahmed, 2015). How do memory T cells retain lineage commitment? It is still an unsolved question. Transcriptional and epigenetic profiling suggest that epigenetic remodeling during effector phase may play an important role in retaining lineage characteristics in memory T_FH_ cells (Wilson et al., 2009; Josefowicz, 2013; Youngblood et al., 2013). In addition, memory B cells conduct immediate antigen presentation to memory T_FH_ cells, inducing rapid re-expression of BCL6 in reactivated T_FH_ cells (Ise et al., 2014), which safeguards the T_FH_-oriented recall responses.

### Longevity of Virus-Specific Memory T_FH_ Cells

Following the clearance of virus in acute viral infection, virus-specific memory CD8^+^ T cells can persist for a very long period at a stable level, whereas virus-specific memory CD4^+^ T cells gradually decay over time (Homann et al., 2001). However, there is much less knowledge about how memory CD4^+^ T cells sustain longevity compared to that of memory CD8^+^ T cells. By transcriptional profiling, Song et al. reveal multiple genetic programs contributed to the longevity of antigen-specific memory CD4^+^ T cells by maintaining T cell quiescence (Song et al., 2020). In a mouse model of acute infection with LCMV, Wang et al. demonstrated that the mTORC2-Akt-GSK3β axis is critical for the longevity of virus-specific memory T_FH_ and T_H_1 cells by protecting these memory cells from ferroptosis, however this signaling axis seems to be non-essential for memory CD8^+^ T cells (Wang et al., 2022c). The tonic mTORC2 activity in virus-specific memory CD4^+^ T cells is sustained by the IL-7 signaling at memory phase, which suggests an important role of IL-7 signaling in memory T_FH_ cell maintenance (Wang et al., 2022c). Given the essential functions of mTOR signaling pathway in regulating cellular metabolism and the divergent role of mTORC2 in memory CD4^+^ T cells and CD8^+^ T cells (Pollizzi et al., 2015), it is reasonable to speculate that there are certain differences either in metabolic features or/and redox homeostasis between virus-specific memory CD4^+^ and CD8^+^ T cells. Paired comparison analysis of memory CD4^+^ and CD8^+^ T cells may provide valuable clues in further understanding memory CD4^+^ T cell longevity.

## Virus-Specific T_FH_ Cells in COVID-19

### T_FH_ Cell Responses in SARS-CoV-2-Infection and Vaccination

Severe Acute Respiratory Syndrome Coronavirus (SARS-CoV-2) is an enveloped single-stranded positive-sense RNA virus accounting for the ongoing pandemic of COVID-19. SARS-CoV-2 genome encodes 4 structural proteins including surface spike (S) glycoprotein, membrane protein (M), envelop protein (E), and nucleoprotein (N). The S protein of SARS-CoV-2 facilitates viral attachment and entry through the engagement with its cognate receptor, angiotensin converting enzyme-2 (ACE2), which is mediated by the receptor binding domain (RBD) within the S1 subunit of S protein (V'Kovski et al., 2021). SARS-CoV-2 infection induces both humoral and cellular immune responses in hosts (Post et al., 2020; Sette and Crotty, 2021; Shrotri et al., 2021). Commonly, neutralizing antibodies are vital for sterilizing immunity of viral infection. Antibodies targeting S protein and RBD are capable of blocking the attachment and entry of SARS-CoV-2 into host cells to prevent COVID-19 infection (Robbiani et al., 2020; Gupta et al., 2021; Wang et al., 2022a; Wang et al., 2022b). The production of neutralizing antibodies targeting SARS-CoV-2 with high magnitude and durability requires potent B cell responses with the help from virus-specific T_FH_ cells. Evidences from non-human primate studies validated that natural infection of SARS-CoV-2 protects rhesus macaques from reinfection, indicating the occurrence of protective immunological memory post SARS-CoV-2 infection (Chandrashekar et al., 2020; Deng et al., 2020). Both two studies verified viral S protein-specific memory CD4^+^ T cells in rhesus macaques after infection of SARS-CoV-2; moreover, Deng et al. further detected the increased viral S protein-specific central memory CD4^+^ T cells in lymph nodes of rhesus macaques post rechallenge of SARS-CoV-2. Given the bimodal differentiation of virus-specific T_FH_ and T_H_1 cells in acute infection, these studies highlight the importance of virus-specific T_FH_ cells in SARS-CoV-2 natural immunity and vaccine-induced immunological protection.

Vaccine candidates that can induce sufficient antibodies targeting S protein and RBD are reckoned to be protective against SARS-CoV-2 infection. Therefore, investigations on SARS-CoV-2-specifc T cell responses, especially the virus-specific T_FH_ cell’s help to cognate B cell, help to accelerate the vaccine testing pipeline and contribute to vaccine development under the pandemic COVID-19. Currently, a plethora of vaccines are applied to fight against COVID-19, including mRNA vaccines, adenoviral vector-based vaccines, recombinant protein vaccines, and inactivated virus vaccines (Grigoryan and Pulendran, 2020). According to the results from clinical trials, all vaccines induce detectable antibodies against SARS-CoV-2. To now, the most efficient vaccine platform is mRNA/LNP, which delivers mRNA encoding S protein of SARS-CoV-2 by lipid nanoparticle (LNP) to host cells and induces robust immune responses towards the S antigen (Laczkó et al., 2020; Painter et al., 2021; Mudd et al., 2022). Immunization with SARS-CoV-2 mRNA vaccines foster potent antigen-specific GC responses (Laczkó et al., 2020) and virus-specific CD4^+^ T cells (Painter et al., 2021) to generate robust neutralizing antibody responses. By analyzing T cells from samples of lymph nodes acquired by fine-needle aspiration from donors who received mRNA vaccines, Mudd et al. further underscored the vital role of virus-specific T_FH_ cell responses in mRNA vaccine-induced robust and durable immunological protection against SARS-CoV-2 (Mudd et al., 2022).

### The Functions of SARS-CoV-2-Specific T_FH_ Cells

In infected individuals, the durable neutralization and memory B cells can be predicted by prompt CD4^+^ T cell responses, especially the strong circulating T_FH_ (cT_FH_) cell responses (Gong et al., 2020; Boppana et al., 2021; Narowski et al., 2022). Nevertheless, the imbalanced humoral and cellular immunity were often observed in COVID-19 patients (Oja et al., 2020; Gao et al., 2021). Among convalescents, stronger antibody responses were observed in individuals experienced a severe COVID-19, compared to those got moderate symptoms or asymptomatic individuals (Chen et al., 2020b; Gudbjartsson et al., 2020; Röltgen et al., 2020). In some severe sick patients, remarkably strong virus-specific IgG responses were observed, along with decreased CD4^+^ T cell responses (Oja et al., 2020). By sequencing the B cell receptor repertoires, Schultheiß et al. further found that individuals who have much severer clinical course got a markedly lower percentage of B cells carrying un-mutated BCRs (Schultheiß et al., 2020), which indicates a profound T_FH_ cell-mediated SHM-and-selection have occurred. Above data suggest a gradually compromise of CD4^+^ T cell responses during COVID-19 progression. Impaired GC reaction was also observed in some cases, of which Naoki Kaneko et al. showed that GC and BCL6^+^ T_FH_ and B cells were absent in lymph nodes and spleens from severely SARS-CoV-2 infected patients who eventually succumbed after admission (Kaneko et al., 2020). Altogether, these findings emphasize the essential role of virus-specific T_FH_ cells in natural immunity to control COVID-19.

Evidences of robust responses of SARS-CoV-2-specific T_FH_ cell responses upon administration of mRNA vaccines suggest that virus-specific T_FH_ cell responses contribute to the successful immunization of this preeminent vaccine platform (Painter et al., 2021; Mudd et al., 2022). Moreover, using T_FH_ cell-deletion (T_FH_-DTR) mice, Cavazzoni et al. showed that reduction of T_FH_ cells results in compromised GC responses and decreased production of anti-S and anti-RBD IgG upon SARS-CoV-2 protein vaccination (Cavazzoni et al., 2022), suggesting that virus-specific T_FH_ cell response is also essential to establish an optimized immune protection in traditional immunization strategy.

On the other hand, T_FH_ cells may also play a pathogenic role in certain circumstances. Wang et al. showed that the hyper-functional CD8^+^ and CD4^+^ T cells were associated with the pathogenesis of extremely severe COVID-19 patients (Wang et al., 2020). In another study conducted by Meckiff et al., the authors found compared to non-hospitalized COVID-19 patients, increased cytotoxic T_FH_ cells which manifest high production of IFN-γ, IL-2, and TNF-α were observed in hospitalized patients with severe illness (Meckiff et al., 2020). In addition, Fenoglio et al. revealed the pathogenic roles of CCR4^+^ and CCR6^+^ T_FH_ cells in COVID-19 patients (Fenoglio et al., 2021). Moreover, it is necessary to pay additional attentions to COVID-19 patients who also suffer from other diseases associated with T_FH_ cell responses, like HIV-infection, autoimmune diseases and cancers treated with immune checkpoint blockade (ICB) therapies (Picchianti-Diamanti et al., 2021; Riou et al., 2021; Salomé and Horowitz, 2021).

### Memory SARS-Cov-2-Specific T_FH_ Cells

Bacher et al. suggested that there are pre-existing memory T cells with low avidity and a cross-reactivity to SARS-CoV-2 in unexposed individuals (Bacher et al., 2020). Given the observation that excessive but low-avidity T cell response to SARS-CoV-2 features the severe COVID-19 but not the mild disease, Bacher et al. questions the protective role of pre-existing cross-reactive memory T cells in anti-SARS-CoV-2 immunity (Bacher et al., 2020). However, other investigations targeting T cell responses to SARS-CoV-2 in unexposed individuals revealed that cross-reactive memory T_FH_ cells could trigger a rapid and superior antibody response to SARS-CoV-2, which might exert better viral control in upper respiratory tract and lung (Grifoni et al., 2020; Lipsitch et al., 2020). The latter view was appreciated by Bonifacius et al., who showed that COVID-19 patients with pre-existing anti-human coronavirus CD4^+^ and CD8^+^ T cells with cross-reactivity of endemic coronaviruses manifested higher frequency of SARS-CoV-2 S protein-specific T cells (Bonifacius et al., 2021). In addition, Mateus et al. observed a significantly higher frequency of viral S protein-specific CD4^+^ T cells and stronger neutralizing antibody responses in vaccinated individuals who present pre-existing SARS-CoV-2 S protein-specific CD4^+^ T cells than subjects with no cross-reactive memory (Mateus et al., 2021). Therefore, besides the classical functions of virus-specific memory T_FH_ cells, the pre-existing virus-specific T_FH_ cells also function to favor T cell and antibody responses against SARS-CoV-2.

Following SARS-CoV-2 infection, substantial virus-specific T cell memory responses are induced in convalescent individuals, whereas the breadth and magnitude were positively correlated to the disease severity of COVID-19 (Peng et al., 2020). The longitude study of T/B cell and antibody responses to COVID-19 revealed a long duration of CD8^+^ and CD4^+^ T cell memory and neutralizing antibodies against SARS-CoV-2 (Dan et al., 2021). In general, accruing evidences suggest a profound protection of nature infection of COVID-19 and vaccination. The emerging incompetent immune protection from vaccination or immunological memory of convalescent individuals is more associated with the rapid evolution of SARS-CoV-2 variants (Thakur et al., 2022; Woldemeskel et al., 2022). SARS-CoV-2 continuously undergoes genetic mutations or viral recombination, resulting in variants with possible differences in transmissibility, clinical manifestation, and immunogenicity. To date, the variant of concern (VOC) is Omicron variant (B.1.1.529), which harbors as many as 36 substitutions in viral S protein and total 59 mutations in whole genome compared with SARS-CoV-2 ancestral strain, leading to immune evasion from neutralization by vaccination- and infection-induced antibodies (Woldemeskel et al., 2022). Although Omicron evade a large fraction of antibodies, its neutralizing antibodies are still represented in a portion of memory B cell repertoire induced by mRNA vaccines (Sokal et al., 2022).

Multiple studies revealed that despite the decay of protective serologic components and decreased effectiveness against infection, vaccines developed based on ancestral strains still efficiently protect individuals from SARS-CoV-2 variants-induced hospitalization and/or severe diseases (Nanduri et al., 2021; Rosenberg et al., 2021; Tenforde et al., 2021). This phenomenon highlights the relative impervious function of T cell immunity against SARS-CoV-2 variants, given that virus-specific T cells mainly act to eliminate infected cells (CD8^+^ T cell) and help B/CD8^+^ T cells responses after activation (CD4^+^ T cell), rather than to directly prevent infection like neutralizing antibodies do. In addition, a recent study demonstrated that the preserved T cell reactivity to variant Omicron variant in most infected and vaccinated individuals can be enhanced shortly after booster vaccination (Naranbhai et al., 2022). In this study, Naranbhai et al. identified about 79% individuals with a preserved T cell reactivity to the viral S protein of Omicron. Moreover, the effector T cell responses to SARS-CoV-2, including both wild type and Omicron strain, were enhanced after additional booster vaccine, accompanied with proliferative memory viral S protein-specific CD4^+^ T cell responses but reduced CD8^+^ T cell responses to Omicron. This evidence indicates that virus-specific memory T cell, especially virus-specific memory CD4^+^ T cell, is a silver lining to the plight of controlling circulating SARS-CoV-2 variants. Notably, heterologous vaccination was used in the abovementioned infection/vaccination-booster stratagem, providing corroborative evidence to the effectiveness of the heterologous sequential vaccination strategy against mutant VOCs of SARS-CoV-2. Compared to homologous vaccination, multiple kinds of heterologous sequential immunization were proved to be superior to induce broad neutralization against VOCs, including combination of inactivated vaccine followed by heterologous mutant RBD vaccine (Song et al., 2022), adenoviral vectored vaccine followed by mRNA vaccine (Pozzetto et al., 2021), inactivated vaccine followed by mRNA vaccine (Zuo et al., 2022), and inactivated vaccine followed by adenoviral vectored vaccine (Li et al., 2022). Given the barely affected memory CD4^+^ T cell responses to peptide pool of Omicron S protein (Naranbhai et al., 2022), it is thus clear that virus-specific memory T_FH_ cells play an important role in the generation of potent and broad neutralizing antibodies to VOCs induced by the heterologous sequential vaccination strategy.

## Concluding Remarks

Since the initial seminal description of T_FH_ cells in 2000 (Breitfeld et al., 2000; Schaerli et al., 2000), many characteristics, functions, and underlying mechanisms of T_FH_ cells have been uncovered over the past two decades. We illustrate the multiple lines and underpinnings of the T_FH_ cell differentiation and maintenance during viral infection in **Figure 1**. But still a lot of puzzles remain to be solved. For example, the extrinsic and intrinsic factors that determine the fate commitment of antigen-specific T_FH_ cells are still unknown. Maybe the differentiation of T_FH_ cells is not a “default” pathway (Choi et al., 2020), and deciphering the networks regulating T_FH_ cell differentiation needs more intense investigations. In addition, how memory T_FH_ cells retain lineage features and prolong over time is fascinating and merits further studies. Better understanding of virus-specific T_FH_ cells will be of great importance for optimizing anti-viral vaccine development, including SARS-CoV-2 vaccines.

## Author Contributions

YW and QT drafted and revised the manuscript with LY. All authors contributed to the article and approved the submitted version.

## Funding

This work was supported by grants from the National Key Research Development Plan (2021YFC2300502), and the National Natural Science Foundation of China (No. 32030041 to LY, No. 31800748 to YW, and No. 31800742 to QT).

## Conflict of Interest

The authors declare that the research was conducted in the absence of any commercial or financial relationships that could be construed as a potential conflict of interest.

## Publisher’s Note

All claims expressed in this article are solely those of the authors and do not necessarily represent those of their affiliated organizations, or those of the publisher, the editors and the reviewers. Any product that may be evaluated in this article, or claim that may be made by its manufacturer, is not guaranteed or endorsed by the publisher.
